# Phage-derived depolymerase targeting the K27 capsule impairs *Klebsiella pneumoniae* virulence, biofilm formation, and promotes immune clearance

**DOI:** 10.1080/22221751.2026.2645857

**Published:** 2026-03-13

**Authors:** Magdalena Pelka, Weronika Czekala, Agnieszka Kwiatek, Marta Polanska, Barbara Maciejewska, Aleksandra Otwinowska, Piotr Golec, Agnieszka Wyszyńska, Zuzanna Drulis-Kawa, Monika Adamczyk-Popławska

**Affiliations:** a Department of Molecular Virology, Institute of Microbiology, Faculty of Biology, University of Warsaw, Warsaw, Poland; b Laboratory of Molecular Microbiology and Genetics, Institute of Biochemistry and Biophysics, Polish Academy of Sciences, Warsaw, Poland; c Department of Animal Physiology, Institute of Functional Biology and Ecology, Faculty of Biology, University of Warsaw, Warsaw, Poland; d Department of Pathogen Biology and Immunology, University of Wroclaw, Wroclaw, Poland; e Department of Bacterial Genetics, Institute of Microbiology, Faculty of Biology, University of Warsaw, Warsaw, Poland

**Keywords:** *Klebsiella pneumoniae*, depolymerase, capsular polysaccharide, antimicrobial therapy, serotype K27

## Abstract

The global rise of multidrug-resistant *Klebsiella pneumoniae* underscores the urgent need for alternative therapeutic strategies. Bacteriophage-derived depolymerases have emerged as promising antimicrobial factors, selectively degrading bacterial capsules and impairing key pathogenic traits. We characterize a novel depolymerase, PRA33gp45, associated with the structural protein of bacteriophage vB_KpnP_PRA33. Bioinformatic structural analyses predicted endo-*N*-acetyl neuraminidase-like activity and canonical depolymerase domain architecture. The recombinant PRA33gp45 specifically hydrolysed capsular polysaccharides (CPS) of K27 serotype *K. pneumoniae* and produced characteristic halo zones on bacterial lawns, confirming its enzymatic activity. Capsule staining demonstrated rapid and progressive capsule degradation within 120 min of treatment. PRA33gp45 significantly inhibited biofilm formation, disrupted mature biofilms, and altered biofilm architecture as visualized by confocal microscopy. Depolymerase pre-treatment markedly reduced *K. pneumoniae* survival within A549 human lung epithelial cells, without exhibiting any cytotoxic effect and sensitized bacteria to complement-mediated killing in human serum. Finally, PRA33gp45 treatment of *K. pneumoniae* lowers morbidity and mortality in the *Galleria mellonella* larvae model. Collectively, these findings identify PRA33gp45 as a novel and highly specific depolymerase that diminishes *K. pneumoniae* virulence by targeting its protective capsule, impairing persistence as biofilm, and enhancing innate immune clearance. Its safety and efficacy suggest potential as an antimicrobial or adjuvant therapeutic agent against K27-type *K. pneumoniae* infections, particularly in the context of multidrug resistance and emerging pathogens.

## Introduction

*Klebsiella* spp. are Gram-negative *Enterobacteriaceae*, part of the normal human gut microbiota [1]. Among them, *Klebsiella pneumoniae* is a significant clinical concern. It colonizes 1% to over 38% of humans asymptomatically (oropharynx or gastrointestinal tract) but also acts as a nosocomial opportunistic pathogen, mainly affecting immunocompromised, hospitalized individuals, neonates, and the elderly [2]. *K. pneumoniae* causes pneumonia, urinary tract infections, septicaemia, meningitis, and acute liver abscesses, contributing to increased mortality [2,3]. It belongs to the ESKAPEE (*Enterococcus faecium*, *Staphylococcus aureus*, *K. pneumoniae*, *Acinetobacter baumannii*, *Pseudomonas aeruginosa*, *Enterobacter* spp., and *Escherichia coli*) group – multidrug-resistant pathogens causing difficult-to-treat infections [4]. Due to high mortality, rapid spread, and resistance to last-resort antibiotics, carbapenem-resistant *K. pneumoniae* is classified by the World Health Organization (WHO) as a Critical Priority Pathogen [5].

The urgent need for new therapies against *K. pneumoniae* infections is well recognized [1,6]. Phage therapy against drug-resistant bacteria has gained renewed interest. Bacteriophages produce enzymes that degrade bacterial extracellular polysaccharides, capsule polysaccharides (CPS), exopolysaccharides (EPS), or lipopolysaccharides (LPS), facilitating viral attachment and infection with high specificity [7–9]. In Gram-negative pathogens, polysaccharides are major virulence factors protecting bacteria from environmental stress and host immunity [10]. These polymers increase resistance to abiotic stress and aid competition. Within biofilms, polysaccharides shield bacteria from toxins, antibiotics, immune response, and viral infection, making phage-derived depolymerases promising anti-biofilm agents [11].

A thick layer of CPS (K-antigen), made of complex acidic polysaccharides, is the main virulence factor of *K. pneumoniae*. Although structures of serotypes K1–K82 are known [12], CPS composition for over 100 capsule types remains uncharacterized [13,14]. CPS protects *Klebsiella* from phagocytosis and serum bactericidal proteins by masking surface antigens, enhancing immune evasion. It also affects bacterial adherence and invasiveness in human cells, as well as colonization and dissemination to organs via the bloodstream [15–17]. Because of its key role in virulence, CPS-degrading enzymes represent promising antibacterial agents [18,19].

This study focuses on the identification of a novel CPS depolymerase, PRA33gp45, associated with the structural protein of bacteriophage vB_KpnP_PRA33 (PRA33). PRA33gp45 was produced as a recombinant protein and confirmed for its ability to hydrolyse specifically K27 serotype CPS. Additionally, we evaluated the effect of PRA33gp45 on biofilm formation and degradation, the interaction of bacteria with human epithelial lung cells, and sensitizing bacteria to human serum. We also demonstrated that PRA33gp45 preincubation or co-administration with *K. pneumoniae* protects *Galleria mellonella* larvae from infection and death in an *in vivo* model.

## Materials and methods

### *In silico* analysis of the genome of vB_KpnP_PRA33 bacteriophage

The sequence of the genome of phage vB_KpnP_PRA33 is deposited at GeneBank (KY652723) [20]. Protein sequences were compared against various databases (Blastp, Conserved Domains from NCBI) and screened for any enzymatic domains (Swiss Model [21], EzyPred [22], HHpred [23], MotifFinder [24], and DePP servers [25]). The structure of chosen proteins was solved by AlphaFold Server [26] and visualized with PyMol [27]. Protein sequence alignment was performed with ClustalW [28]. A phylogenetic tree was generated with FastTree [24].

### Bacterial strains

*E. coli* Top10 F’ cells (Thermo Fisher Scientific, Waltham, USA) were used for molecular cloning and T7 Express (New England Bio Labs, Ipswich, MA, USA) for recombinant protein overexpression.

*K. pneumoniae* A31_1, isolated from animal infection, was used in all experiments unless otherwise stated.

Bacteria from different species (Table S1) and collection of *K. pneumoniae* capsular serotype strains (Table S2) were used for PRA33gp45 specificity testing.

*Klebsiella* spp. K-type panel used in this study originates from the Collection de l’Institut Pasteur (CIP), Paris, France; the National Collection of Type Cultures (NCTC), the UK Health Security Agency (UKHSA); the collection of the Department of Pathogen Biology and Immunology, Wroclaw, Poland; and the *Klebsiella* Acquisition Surveillance Project at Alfred Health (KASPAH), Melbourne, Australia [29], provided by Kath Holt at the London School of Hygiene and Tropical Medicine (LSHTM), Department of Infection Biology, London, UK. The full K-type panel includes 134 strains representing 119 distinct serotypes. Bacteria were cultured in Tryptone Soya Broth, Agar (TSB or TSA, Oxoid, Thermo Fisher Scientific, Waltham, MA, USA), or Luria–Bertani broth (LB, Biomaxima, Lublin, Poland) at 37 °C.

### Determination of the host range and antimicrobial activity of phage PRA33-derived proteins

Spot tests were conducted on LB agar plates with 0.7% top agar overlaid containing bacteria (Table S1). Unpurified extracts or purified PRA33gp45 were spotted onto the bacterial lawns. After 24 h at 37 °C, plates were checked for lytic zones. Appropriate buffers served as negative controls.

### Depolymerase activity screening against *Klebsiella* serotype collection

*Klebsiella* (Table S2) was spread evenly onto agar plates. Excess liquid was removed after 3 min, and the plates were air-dried. Then, crude PRA33gp45 extract or control buffer (0.5 M NaCl, 20 mM NaH_2_PO_4_, pH 7.4) was spotted onto plates. After 24 h, the presence of lytic or semi-clear zones with halo formation within the bacterial lawn suggested a depolymerase activity.

### Serotyping of *K. pneumoniae* strain A31_1

Serotyping of *K. pneumoniae* strain A31_1 was performed by PCR (Tables S3 and S4) targeting variable regions of the *wzc* gene [30] using the A31_1 genome as a template. PCR products were sequenced and analysed with BLASTn.

### Microtiter-plate adherence biofilm assay

The microtiter-plate adherence assay was performed as described [31,32].

*K. pneumoniae* A31_1 cultured to an OD_600_ = 0.2 was mixed with purified PRA33gp45 depolymerase (1:1) at final concentrations ranging from 0.14 to 700 nM. After 24 h, non-adherent cells were removed by extensive washing, and biofilms stained with 0.1% crystal violet (CV). Bounded dye was solubilized with 30% acetic acid, transferred to a fresh plate, and biofilm was quantified by absorbance measurements using an automated Sunrise microplate reader and Magellan software (Tecan).

To assess the effect of depolymerase on established biofilms, 24-h biofilms (formed from bacterial culture at OD_600_ = 0.2) were treated with PRA33gp45 (70–700 nM) for 2 h, followed by quantification as above. Negative controls contained bacteria, LB, and buffer.

### Pre-treatment of *K. pneumoniae* cells with PRA33gp45 depolymerase

*K. pneumoniae* A31_1 was cultured until reaching an OD_600_ of 0.4–0.5. The cells were then harvested by centrifugation (6000 × *g*, 10 min) and resuspended in F-12K medium to an OD_600_ of 1.7–1.75. This suspension was incubated with varying concentrations of purified PRA33gp45 for 90 min at 37 °C.

### Adhesion and invasion of human cells

Assays were adapted from previous works [31–34]. The human lung epithelial cell line A549 (ATCC CCL-185) was cultured in F-12K medium (BioWest, Nuaille, France) supplemented with 10% foetal bovine serum (Cytogen, Zgierz, Poland) at 37 °C in 5% CO_2_. Cells were grown in 24-well plates to confluence.

Fresh F-12K medium was added to bacteria pre-treated with 1000, 700, 350, or 35 nM PRA33gp45, and the resulting suspensions were applied to A549 cells with multiplicity of infection of 30. After 2 h, cells were washed to remove non-adherent bacteria, and A549 cells were lysed with 0.1% Triton X-100 for 10 min. Cell-associated CFUs were enumerated by plating serial dilutions. For invasion assays, extracellular bacteria were killed by gentamicin (100 µg/ml) for 45 min prior to lysis with Triton.

The total CFU was calculated as the sum of CFUs from the supernatant and lysates. Adhesion index was defined as cell-associated CFU divided by total CFU [31], and the invasion index was defined as gentamicin-resistant CFU divided by corrected cell-associated CFU [34].

### *Galleria mellonella in vivo* assay

Infection of *G. mellonella* with *K. pneumoniae*, as well as the estimation of the effect of PRA33gp45, was performed as previously described with minor modifications [18]. Briefly, larvae were infected by injection with 3.5 × 10^6^ CFUs of: (i) untreated bacteria in dialysis buffer, (ii) bacteria preincubated for 90 min at 37 °C with PRA33gp45 (700 or 1400 nM), and (iii) bacteria administered together with PRA33gp45 (700 or 1400 nM). Control groups included: uninfected larvae, larvae injected with dialysis buffer, and larvae injected with enzyme (700 nM). Larvae mobility, melanisation and survival were recorded for 72 h post-injection with 24 h intervals. Each test was performed in four independent experiments with 10 larvae per trial.

### Sensitivity of *K. pneumoniae* to the bactericidal effect of human serum complement

*K. pneumoniae* A31_1, treated with 1400 nM PRA33gp45 as described above, were mixed 1:1 (v/v) with 50% pooled human serum from healthy volunteers (25–60 years old, with no known immune disorders), collected with informed consent following institutional guidelines. After incubation at 37 °C for 3 h under gentle rotation, surviving bacteria were enumerated. Cells preincubated in dialysis buffer served as controls. To verify complement involvement, human serum was heat-inactivated at 56 °C for 30 min. Viable bacteria were expressed as CFU/ml.

### Statistics

All experiments were performed at least in triplicate, and results were averaged. Normality was assessed by the Shapiro–Wilk test. For normally distributed data, one-way ANOVA, Brown-Forsythe, and Welch’s tests were used to compare groups, followed by Dunnett’s T3 post-hoc test for pairwise comparisons. Non-normal data were analysed by the Kruskal–Wallis test with Dunn’s post-hoc test. *G. mellonella* survival curves were plotted using the Kaplan– Meier method, and analysed by using the log-rank Mantel–Cox with GraphPad Prism 11.0.0. Statistical significance was set at *p* < 0.05.

Molecular cloning, protein purification, cytotoxicity assessment, isolation of capsule polysaccharides, zymogram assay, Maneval’s staining, immunostaining, and confocal microscopy (SCLM) are described in the Supplementary Material.

## Results

### Functional bioinformatic analysis of bacteriophage vB_KpnP_PRA33 structural proteins

*Przondovirus* vB_KpnP_PRA33 (PRA33) was selected due to its ability to produce, typical for virion-associated polysaccharide-degrading depolymerases, clear lytic plaques with translucent halos on *K. pneumoniae* A31_1 lawns [7,20,35]. We focused on ORFs encoding tail fibre or spike-like structural proteins (Table 1). ORF45 encodes a protein similar to the T7 phage *E. coli* tail fibre with known depolymerase activity [7,36]. Analysis predicted putative endo-*N*-acetyl neuraminidase activity and revealed an intramolecular chaperone domain, both typical of depolymerases [37] (Table 1).

**Table 1.** Overview of predicted functions of bacteriophage PRA33 proteins identified by bioinformatic tools.

Protein	Length (aa)	Mass (kDa)	Potential function						
			BV-BRC	BLASTp (e-value)	HHPred (e-value)	Motif Finder (e-value)	Swiss Model (sequence identity)	EzyPred Layer of prediction	DePP (score)
PRA33gp39	192	21.4	Phage non-contractile tail tubular protein Gp11	Tail tubular protein A, 1.33 e-98	Tail tubular protein Gp11, 7.2 e-41	Tail tubular protein A, 1 e-98	Tail tubular protein A of *K. pneumoniae* bacteriophage KP32 98.96%	Not an enzyme 1st layer	0.29
PRA33gp40	791	88.9	Phage non-contractile tail tubular protein Gp12	Tail protein, 0	Phage tail tubular protein B, 5.5 e-115	–	Tail tubular protein B *Klebsiella* phage Kp9 96.46%	Glycosylase 3rd layer	0.94
PRA33gp44	1320	142.31	Phage DNA ejectosome component Gp16, peptidoglycan lytic exotransglycosylase (EC 4.2.2.n1)	Internal virion protein with endolysin domain, 0	Peptidoglycan transglycosylase gp16, 3.5 e-127	Transglycosylase SLT domain, 4 e-31	Peptidoglycan transglycosylase gp16 core proteins of mature T7 66.57%	Not an enzyme 1st layer	0.86
PRA33gp45	1242	135.9	Phage non-contractile tail fibre protein Gp17	Tail fibre protein, 2.22 e-39 N-acetyloneuraminidase	Tail spike protein, 9.4 e-18	Tail fibre protein, 2 e-39	Neck appendage protein, intramolecular chaperone 45.54%	Not an enzyme 1st layer	0.97

**Figure 1.**
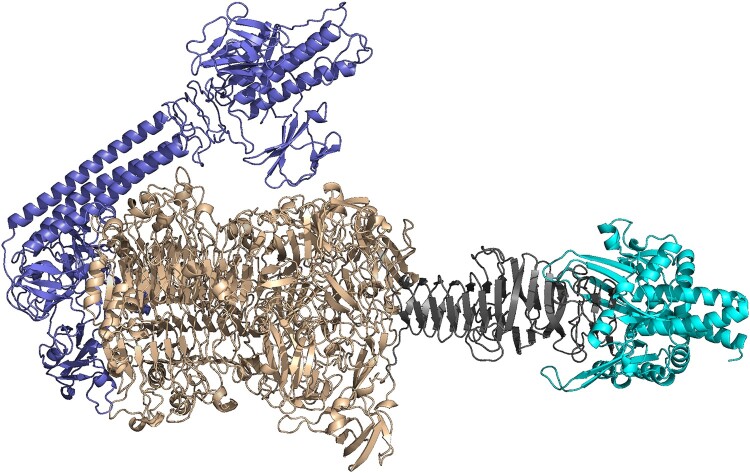
Modular organization of PRA33gp45 (AlphaFold) reflecting a structure of subclass 4B of *Klebsiella* phage depolymerases [39]. Blue, the virion-anchoring N-terminal domain (1–296 aa); beige, the catalytic αH-supplemented β-helix (297–1013 aa); grey, the tail fibre- like extension (1014–1126 aa); cyan, the chaperone-assisted C-terminus (1126–1242 aa).

Three proteins scored highly in the DePP predictor (>0.75), but only PRA33gp45 displayed the 3D structure of the *Klebsiella*-specific depolymerase (Figure 1 and Figure S1). PRA33gp45’s domain architecture includes a canonical N-terminal anchoring domain (1–296 aa) for virion attachment, an αH-supplemented β-helical core (297–1013 aa) forming the enzymatic scaffold, a tail fibre- like domain (1014–1126 aa), and a C-terminal chaperone module (1126–1242 aa) essential for folding and trimer stabilization. According to DepoCatalog, PRA33gp45 is classified as Class 4B (αH-central domain and tail fibre domain) [38].

PRA33gp45 exhibited high amino acid identity (97.58–98.63%) to tail fibre proteins from various *K. pneumoniae* phages (e.g. KpV768 (XXK85098.1); vB_KpnP_BIS33 (YP_009787496.1); vB_KpnP_MUC100 (WOZ56289.1); vB_KpnP_IL33 (YP_009787550.1); vB_Kpn_K27PH129C1 (CAK6597027.1)), and clustered closely with them in phylogenetic analysis (Figure S2). 3D structure comparison yielded high TM-scores (0.827–0.942), confirming their strong evolutionary and structural similarity, though their enzymatic depolymerase activity remains unconfirmed experimentally.

### *In vitro* identification of PRA33gp45 as a putative CPS depolymerase of phage PRA33

**Figure 2.**
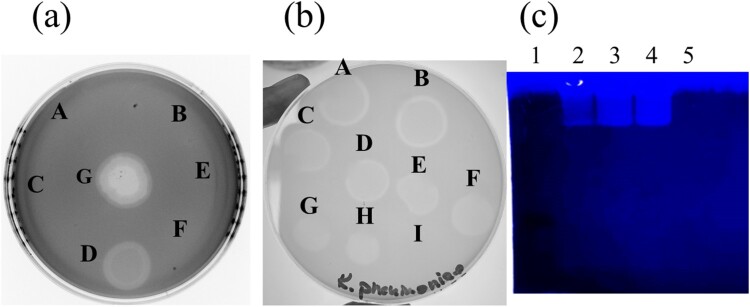
Demonstration of PRA33gp45 depolymerizing activity. (a) Crude extracts of *E. coli* expressing (A) PRA33gp39, (B) PRA33gp40, (C) PRA33gp44, and (D) PRA33gp45 were spotted onto *K. pneumoniae* strain A31_1 lawn. Extracts from cells harbouring the non-recombinant pET28(+) vector (E) or sonication buffer (F) were used as negative controls. (G) Phage PRA33 was used as positive control. (b) Purified PRA33gp45 was applied onto *K. pneumoniae* A31_1 lawn: (A) 1400 nM, (B) 700 nM, (C) 140 nM, (D) 70 nM, (E) 14 nM, (F) 7 nM, (G) 3.5 nM, and (H) 1.25 nM. Dialysis buffer was used as a negative control (I). (c) Zymogram assay performed with CPS isolated from *K. pneumoniae* A31_1. Lane 1: protein molecular marker; lane 2: 1 µg of PRA33gp45; lane 3: 5 µg of PRA33gp45; lane 4: 10 µg of PRA33gp45; lane 5: 5 µg of BSA.

Spot tests, using unpurified extracts of PRA33gp39, PRA33gp40, PRA33gp44, and PRA33gp45, on *K. pneumoniae* A31_1 lawns showed that only PRA33gp45 produced a clear lytic zone with a surrounding translucent halo, mirroring the effect of bacteriophage PRA33 (Figure 2(a)). No lytic activity or halo was observed for other proteins or controls.

Purified PRA33gp45 confirmed depolymerase activity on *K. pneumoniae* A31_1 lawns using serial dilutions (1.25–1400 nM), with a clear halo appearing even at 7 nM (Figure 2(b)). Extended incubation of plates at room temperature enhanced halo size, supporting depolymerase activity of PRA33gp45. Zymogram assay confirmed enzyme hydrolytic activity, as CPS isolated from *K. pneumoniae* A31_1 showed clearing bands at ∼140 kDa, corresponding to the PRA33gp45's molecular weight (Figure 2(c)).

### The surface K27 capsule serves as the primary receptor for PRA33gp45 depolymerase

Extracts containing PRA33gp45 were tested by spotting on lawns of a collection of *Klebsiella* strains (Table S2). Halo formation was observed when crude extract containing PRA33 gp45 was spotted on *K. pneumoniae* CIP 52.232 (serotype K27). This specificity was further confirmed using purified PRA33gp45 protein at different concentrations, which consistently produced the halo effect on the K27 capsular type strain (Figure S3).

The K27 capsular type of *K. pneumoniae* A31_1 was confirmed by *wzc* gene sequencing (Tables S3 and S4), with all PCR products matching K27 serotype (GenBank: AB924565.1).

No lytic zones or halos were observed on various Gram-positive and -negative lawns of bacteria (Table S1), confirming the specificity of the PRA33gp45 depolymerase exclusively for the K27 capsular type of *K. pneumoniae*.

### Phage PRA33-derived depolymerase degrades the capsule of living *K. pneumoniae* A31_1 cells

**Figure 3.**
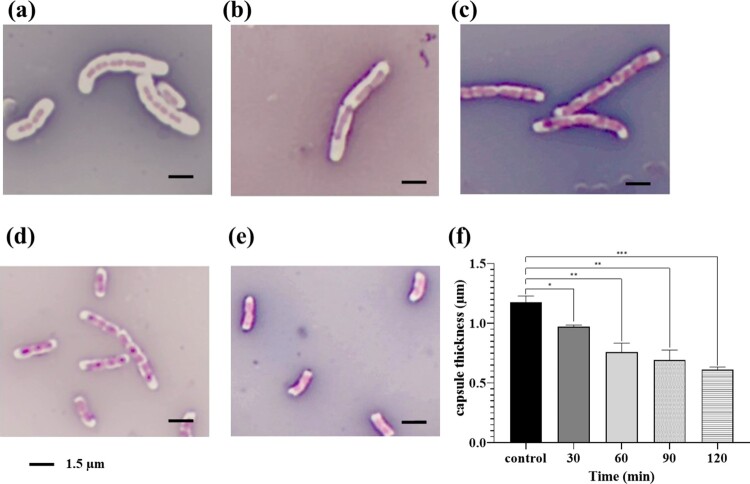
Maneval’s staining of *K. pneumoniae* A31_1 cells. Control cells incubated with PBS (a) or cells treated with 700 nM PRA33gp45 depolymerase for (b) 30 min, (c) 60 min, (d) 90 min, and (e) 120 min. (f) Capsule thickness in µm measured by ImageJ (***: *p* < 0.001, **: *p* < 0.01, *: *p* < 0.05).

In control samples, live *K. pneumoniae* A31_1 cells were surrounded along their entire length by a prominent capsular layer, visible as a thick white halo around the pink-stained cells (Figure 3). In contrast, a progressive reduction in capsular material was observed in purified PRA33gp45 depolymerase-treated cells after 60 and 90 min. Following 120 min of treatment, cells appeared almost entirely devoid of capsules, with only faint capsule remnants remaining mainly at the bacterial poles, and cell aggregates were no longer observed. Capsule thickness measurements confirmed a progressive decrease, dropping from 1.175 ± 0.05 to 0.61 ± 0.02 µm after 120 min incubation with PRA33gp45 (Figure 3(f)).

### PRA33gp45 depolymerase impedes biofilm formation by *K. pneumoniae*

**Figure 4.**
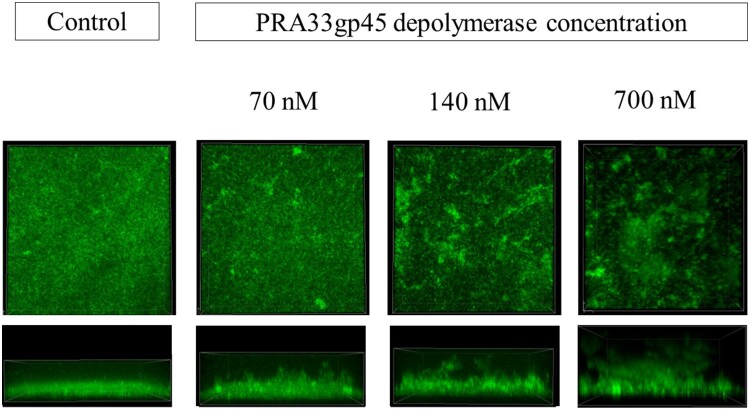
Scanning confocal laser microscopy (SCLM) images of *K. pneumoniae* A31_1 biofilms. Cells were incubated with PRA33gp45 depolymerase and allowed to form biofilms on abiotic surfaces for 20 h.

As observed by SCLM, in the absence of PRA33gp45, a homogeneous and confluent biofilm extensively and uniformly covered the substrate (Figure 4). The presence of PRA33gp45 (70, 140, or 700 nM) disrupted biofilm structure, resulting in heterogeneous coverage with cell-free areas. PRA33gp45 enhanced the appearance of prominent vertical protrusions resembling detaching biofilm fragments. The maximum and average thickness as well as biofilm volume increased in the presence of PRA33gp45, while the surface area ratio decreased compared to parameters determined for control biofilm (Table 2).

**Table 2.** Representative quantitative biofilm structural parameters obtained using COMSTAT 2.0 analysis of images acquired by confocal laser scanning microscopy (SCLM).

Depolymerase PRA33gp45	–	+	+	+
Depolymerase concentration (nM)	0	70	140	700
Biofilm volume (µm^3^/µm^2^)	66.77	98.74	104.51	131.57
Biofilm maximum thickness (µm)	80	108	113	153
Biofilm average thickness (µm)	39.95	53.98	56.48	76.49
Biofilm surface area (µm^2^/µm^3^)	1.68	1.11	1.04	0.84

**Figure 5.**
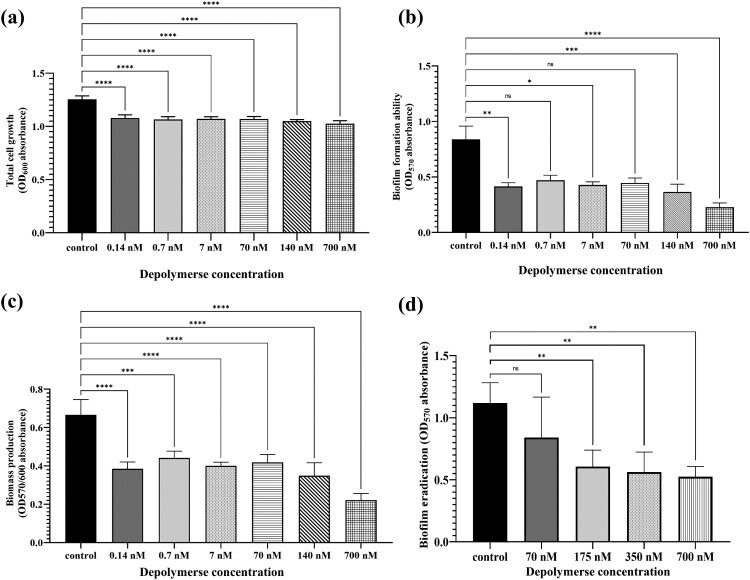
PRA33gp45 depolymerase-mediated inhibition of *K. pneumoniae* A31_1 biofilm. Biofilm formation was analysed by CV staining. (a) Total cell growth (biofilm-forming and planktonic cells) was measured at OD_600_; (b) Biofilm biomass formation ability was measured at OD_570_; (c) Biofilm biomass production: the ratio of cells that form biofilm (OD_570_) versus planktonic cells (OD_600_); (d) Biofilm biomass after treatment of 24-h bacterial biofilm with PRA33gp45 for 2 h (OD_570_). Asterisks represent statistically significant differences in comparison to the control strain (****: *p* < 0.001, ***: *p* < 0.003, **: *p* < 0.005, *: *p* < 0.02; ns, non-statistically significant). Control: *K. pneumoniae* treated by dialysis buffer only.

Biofilm establishment was then examined by CV staining [31,39]. Presence of PRA33gp45 resulted in about 18% reduction in overall bacterial cell density (OD_600_) (Figure 5(a)). Biofilm formation (OD_570_) was inhibited by PRA33gp45 at concentrations of 140 and 700 nM (Figure 5(b)).

Notably, biomass analysis (OD_570/600_ ratio) confirmed depolymerase inhibition of biofilm formation across all concentrations (Figure 5(c)), with 700 nM PRA33gp45 reducing biofilm by 67.16% and 0.14 nM reducing it by 43.28% relative to untreated *K. pneumoniae*.

The biofilm-degrading activity was also assessed by treating 24-h-old biofilms for 2 h with purified PRA33gp45 and CV staining (Figure 5(d)). Treatment with 175, 350, and 700 nM of PRA33gp45 led to a significant decrease in biofilm biomass, as compared to untreated control (*p* < 0.01).

### PRA33gp45 modulates the interaction of *K. pneumoniae* with human lung cells

**Figure 6.**
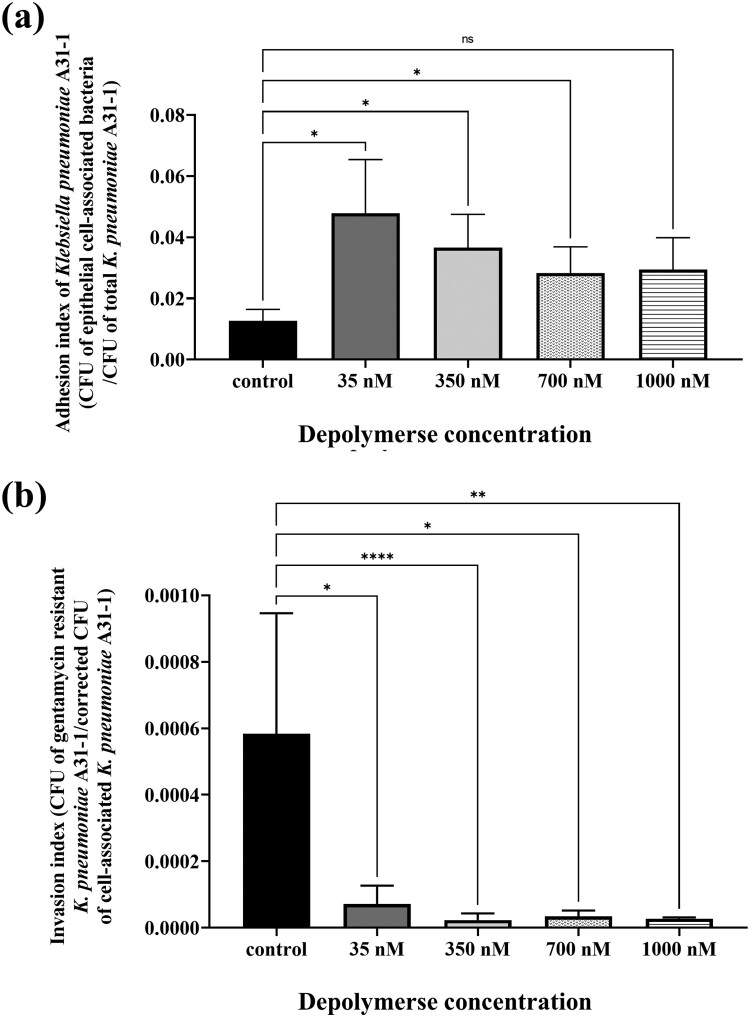
Effect of pre-treatment of *K. pneumoniae* A31_1 with PRA33gp45 on adhesion and invasion of human cells. Bacterial cells were pre-treated with purified PRA33gp45 for 90 min before the addition to human A549 lung epithelial cells. (a) Adhesion indexes and (b) invasion indexes were calculated by CFU/ml enumeration (****: *p* < 0.0001, **: *p* < 0.01, *: *p* < 0.05, ns, non-statistically significant).

**Figure 7.**
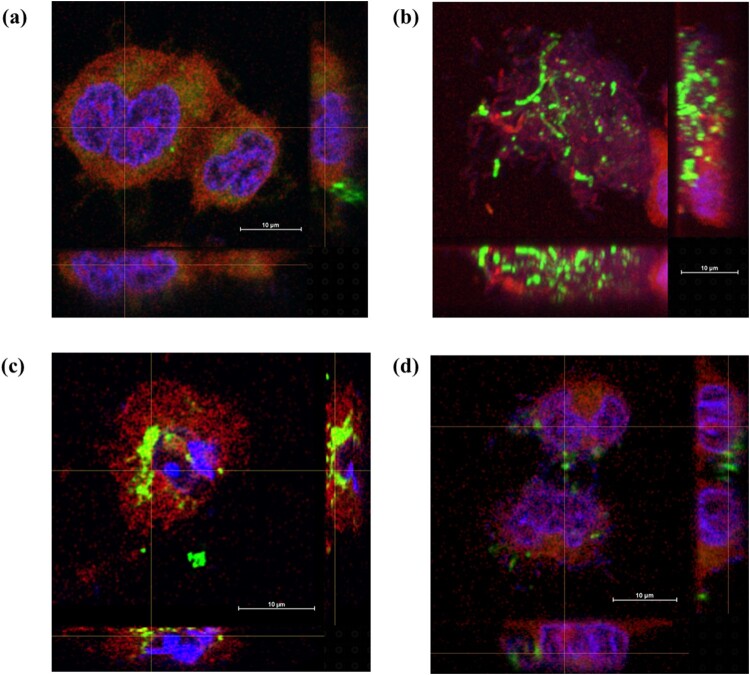
Adhesion and invasion of *K. pneumoniae* A31_1 to human A549 lung epithelial cells, detected by immunostaining with FITC-conjugated pan-anti-*Klebsiella* antibodies and SCLM. (a, c) Human cells infected with untreated bacteria; (b, d) human cells infected with bacteria pre-treated with PRA33gp45. In (c, d), extracellular bacteria were killed by gentamicin treatment.

Human lung epithelial A549 cells were exposed to *K. pneumoniae* A31_1 that had been pre-treated with purified PRA33gp45. Pre-treatment markedly enhanced bacterial adhesion to human lung cells (Figure 6(a)). The adhesion indexes of pre-treated bacteria increased 2.26–3.69 times when compared to depolymerase-unpretreated cells. In contrast, PRA33gp45 reduced bacterial internalization into human lung cell cytoplasm (Figure 6(b)). Pre-treatment with PRA33gp45 at concentrations of 35, 350, 700, and 1000 nM reduced the invasion index by approximately 88%, 96%, 94%, and 95%, respectively. Visualization of bacteria using FITC-conjugated pan-anti-*Klebsiella* antibodies provided deeper insights into host–pathogen interactions (Figure 7). Compared to untreated bacteria (Figure 7(a)), PRA33gp45-treated *K. pneumoniae* showed increased adhesion indexes due to enhanced attachment, followed by rapid penetration into human epithelial cells (Figure 7(b)). However, PRA33gp45-pre-treated bacteria were rarely detected intracellularly after an additional 45 min (Figure 7(d)), indicating that pre-treatment impairs their intracellular survival compared to untreated controls (Figure 7(c)).

Under studied conditions, PRA33gp45 was not cytotoxic to human A549 cells (Figure S4).

### PRA33gp45 sensitized *K. pneumoniae* to human serum

**Figure 8.**
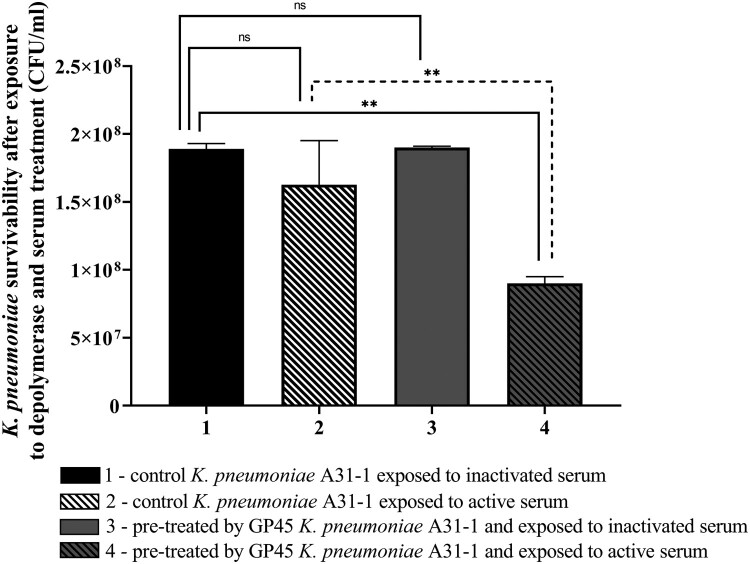
Bactericidal effect of human serum on *K. pneumoniae* A31_1 cells treated with PRA33gp45 depolymerase. ** *p* < 0.006; ns, non-statistically significant.

Exposure of *K. pneumoniae* to 25% human serum did not cause a significant bactericidal effect under the tested conditions (Figure 8). Pre-treatment with PRA33gp45 followed by incubation with non-heat-inactivated serum significantly decreased bacterial survival by 45% (*p* < 0.006), indicating that PRA33gp45 enhances *K. pneumoniae* susceptibility to the bactericidal activity of human serum probably through a complement-dependent mechanism.

### PRA33gp45 prolongs the survival of *K. pneumoniae*-infected *G. mellonella* larvae

**Figure 9.**
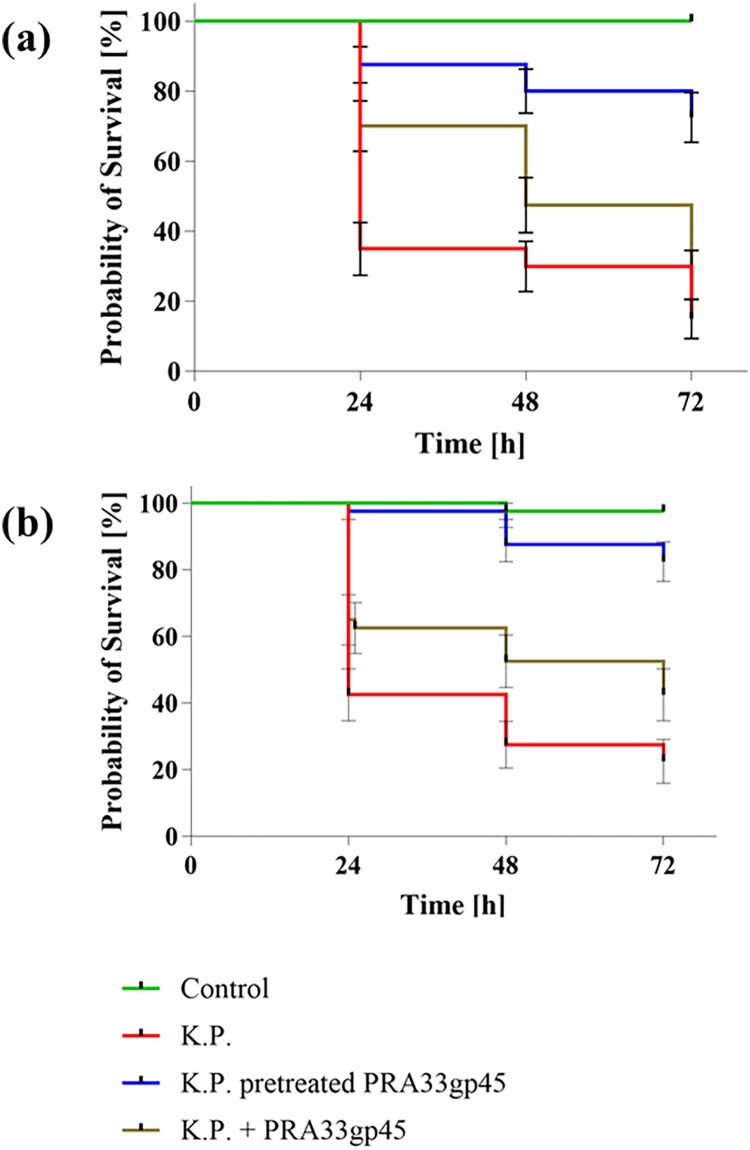
Effect of PRA33gp45 depolymerase on *G. mellonella* larvae survival after *K. pneumoniae A31_1* infection. Larvae received bacteria (K.P.), bacteria preincubated with 700 nM (a) or 1400 nM (b) PRA33gp45, or enzyme (700 nM [a] or 1400 nM [b])co-administered with bacteria. Survival was monitored for 72 h post-infection, with controls (uninfected, buffer-injected, depolymerase-only) showing ∼100 % survival. Data represent 4 independent experiments (total 40 larvae/group); statistical differences were analysed by Mantel-Cox test (*P* < 0.0001).

Following infection with *K. pneumoniae* A31_1 (3.5 × 10^6^ CFUs), only ∼40% of larvae survived within 24 h and ∼25% by 72 h (Figure 9). Preincubation of injected bacteria with 700 nM (Figure 9(a)) or 1400 nM (Figure 9(b)) of purified PRA33gp45 depolymerase increased larval survival to 80% and 90%, respectively, at 72 h post-infection. Co-administration of depolymerase with bacteria also reduced larvae mortality, with >40% survival at 700 nM and ∼50% at 1400 nM by 72 h. We also observed changes in larval melanisation and mobility under the studied conditions. Our findings indicated improved health and higher survival rates in larvae receiving PRA33gp45 supplementation, either as pre-treatment or simultaneously (Figure S5).

## Discussion

Phage-borne depolymerases catalyse the degradation of bacterial exopolysaccharides, which constitute a critical virulence factor, underpinning pathogenic bacterial capacity to evade host immune responses and establish persistent infections.

Numerous studies have described the therapeutic potential of *Klebsiella*-specific depolymerases, primarily against prevalent serotypes like K1, K2, K3, K34, K47, or K64 [11,18,40–45]. Given the extensive diversity of over 100 *K. pneumoniae* capsular types, discovering depolymerases targeting emerging or less-studied serotypes remains critical.

We identified a novel phage-derived protein, PRA33gp45, as possessing hallmark depolymerase features, including conserved catalytic domains, motifs linked to CPS degradation, a canonical 3D depolymerase structure [38] and targeting K27 capsular serotype. To our knowledge, only the K27dep depolymerase (from phage vB_KpnM-20) has been associated with K27 serotype, but its effects on virulence mechanisms were not characterized [44]. K27dep is phylogenetically distant from PRA33gp45, indicating that PRA33gp45 is a distinct, previously undescribed enzyme (Figure S2). We confirmed PRA33gp45 as a functional depolymerase and a promising antimicrobial candidate, effectively disarming key *K. pneumoniae* K27 virulence factors.

Biofilms commonly form on medical devices, increasing patient infection risk [46]. The capsule enables initial substratum coverage and proper biofilm architecture [47]. Capsule mutants show impaired biofilm formation, with unencapsulated *Klebsiella* producing more spread-out, less dense, diffuse biofilms due to weaker bacterial interactions [47]. Thus, capsule degradation may improve outcomes in device-related infections. CV staining and SCLM provided a comprehensive understanding of the impact of CPS hydrolysis by PRA33gp45 on bacterial biofilm. CV staining quantified biofilm biomass, while SCLM visualized live biofilm’s 3D architecture. PRA33gp45-treated bacteria showed poor substratum adhesion, forming taller but less dense biofilms, with reduced biomass. PRA33gp45 also significantly reduced the biomass of 24-h-old biofilms, indicating its potential to eradicate established biofilms. Importantly, depolymerase-mediated biofilm disruption may enhance antibiotic penetration and efficacy as a valuable adjunct to conventional chemical and mechanical biofilm removal strategies, ultimately improving patient outcomes and reducing device-related infections.

*K. pneumoniae* evades host immunity by persisting intracellularly in non-immune cells, promoting dissemination and chronic infection [48,49]. Capsule-deficient strains typically show enhanced adhesion due to exposed adhesins, though effects vary by strain and growth conditions [50–53]. Depolymerase treatment of *K. pneumoniae* is expected to alter interactions with human cells, though evidence remains limited. Our CFU/ml counts and immunostaining visualization showed that PRA33gp45-pre-treated bacteria attach better to A549 lung cells but exhibit strongly impaired intracellular survival, consistent with other findings indicating that depolymerases reduce *Klebsiella* viability in macrophages [18]. This decreased intracellular persistence holds key therapeutic value by limiting the bacteria's presence in the infected organism.

We also demonstrated that *K. pneumoniae* A31_1 is naturally serum resistant, but incubation with depolymerase PRA33gp45 increased its susceptibility to human serum, suggesting the involvement of the complement cascade. This view aligns with previous findings showing that depolymerase treatment of *K. pneumoniae* serotypes K3 and K21 increased bacterial susceptibility to complement-mediated killing [18].

In the *in vivo* model, PRA33gp45 significantly prolonged *G. mellonella* survival post-*K. pneumoniae* infection, with preincubation boosting larvae survival to 80–90% and co-administration yielding 40–50% after 72 h post-infection. This invertebrate model is particularly valuable as the innate immune response of *G. mellonella* larvae resembles that of mammals [54]. Our findings thus support PRA33gp45’s therapeutic promise for combating K27 infections in higher-order organisms.

In conclusion, we provide direct functional evidence that phage-borne PRA33gp45 depolymerase specifically degrades the K27 serotype capsule, thereby reducing *K. pneumoniae* virulence. Our findings underscore its therapeutic potential and safety as an anti-*Klebsiella* agent. Although K27 serotype is not prevalent in human infections, it has triggered significant nosocomial outbreaks, such as pandrug-resistant strains in a 2025 Mexican tertiary-care facility [55]. This underscores the need for targeted antimicrobials against the emerging human pathogen K27 serotype [55,56].

Further studies are required to fully characterize PRA33gp45’s biochemical properties and evaluate its stability, with preliminary indicators such as increasing halo zones in spot tests suggesting favourable resilience under diverse conditions.

## Supplementary Material

FigureS5.tif

Supplementary Methods.docx

Figure S4.TIF

Table S1 S2.docx

Supplementary Results.docx

Figure S1.TIF

Biofilm raw data.xlsx

Figure S2.TIF

Table S4 S5.docx

Table S3.docx

Figure S3.TIF
