# Supplementary figures and images for: Phage-derived depolymerase targeting the K27 capsule impairs Klebsiella pneumoniae virulence, biofilm formation, and promotes immune clearance
Source: Emerg Microbes Infect. 2026 Mar 13;15(1):2645857. doi: 10.1080/22221751.2026.2645857 (PMC13063336; doi:10.1080/22221751.2026.2645857)

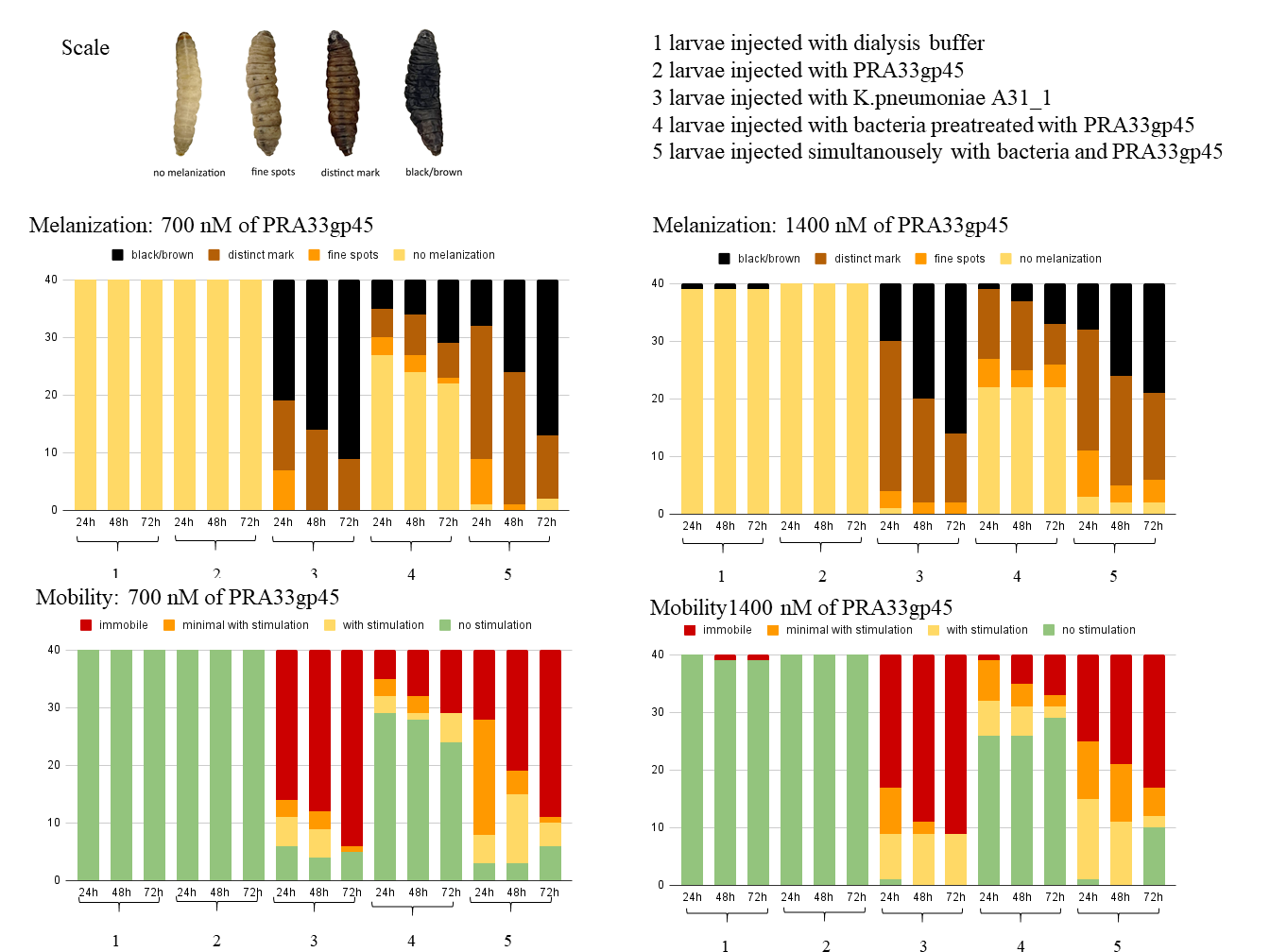

Supplement: FigureS5.tif [file TEMI_A_2645857_SM5225.tif]

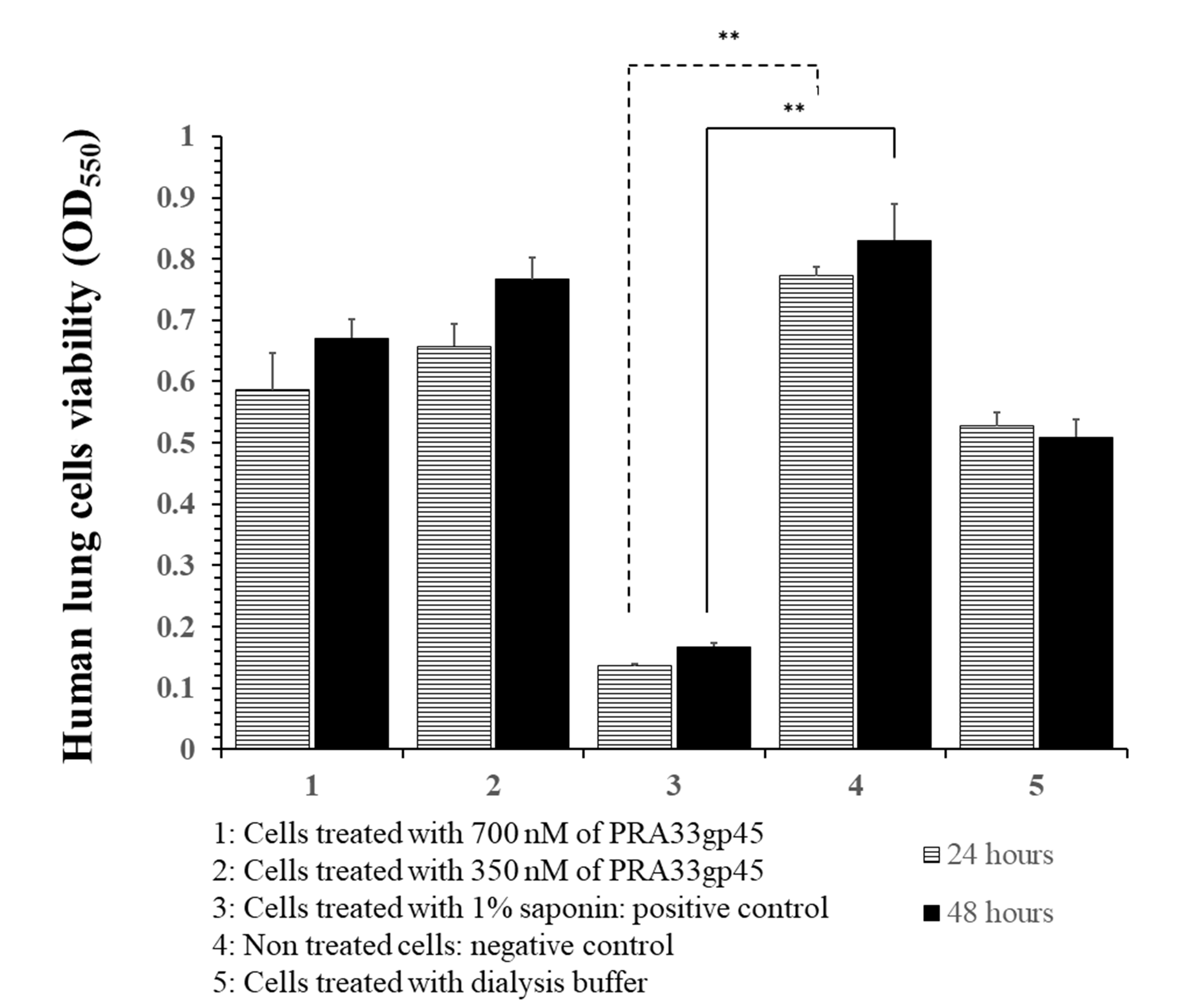

Supplement: Figure S4.TIF [file TEMI_A_2645857_SM5223.tif]

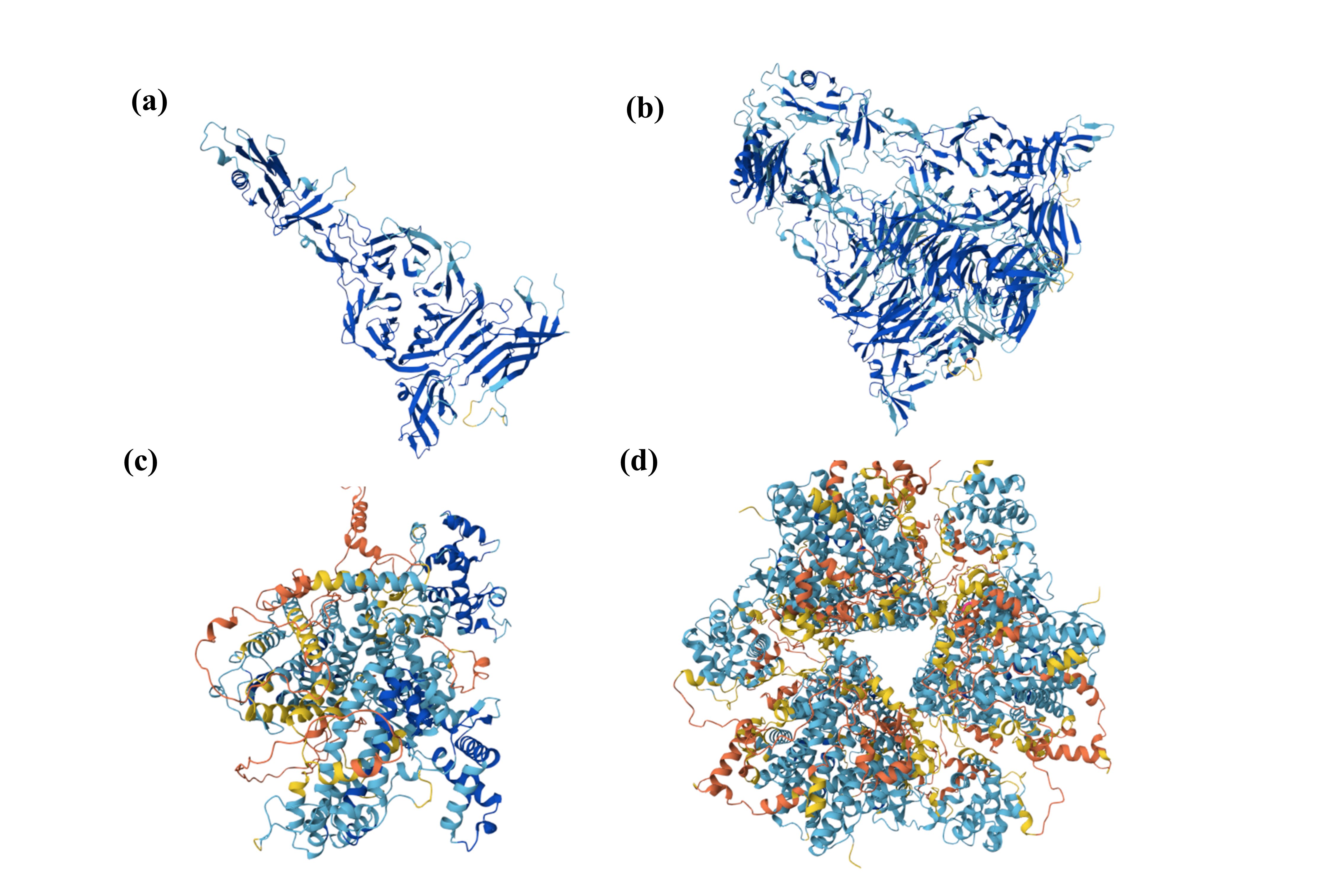

Supplement: Figure S1.TIF [file TEMI_A_2645857_SM5220.tif]

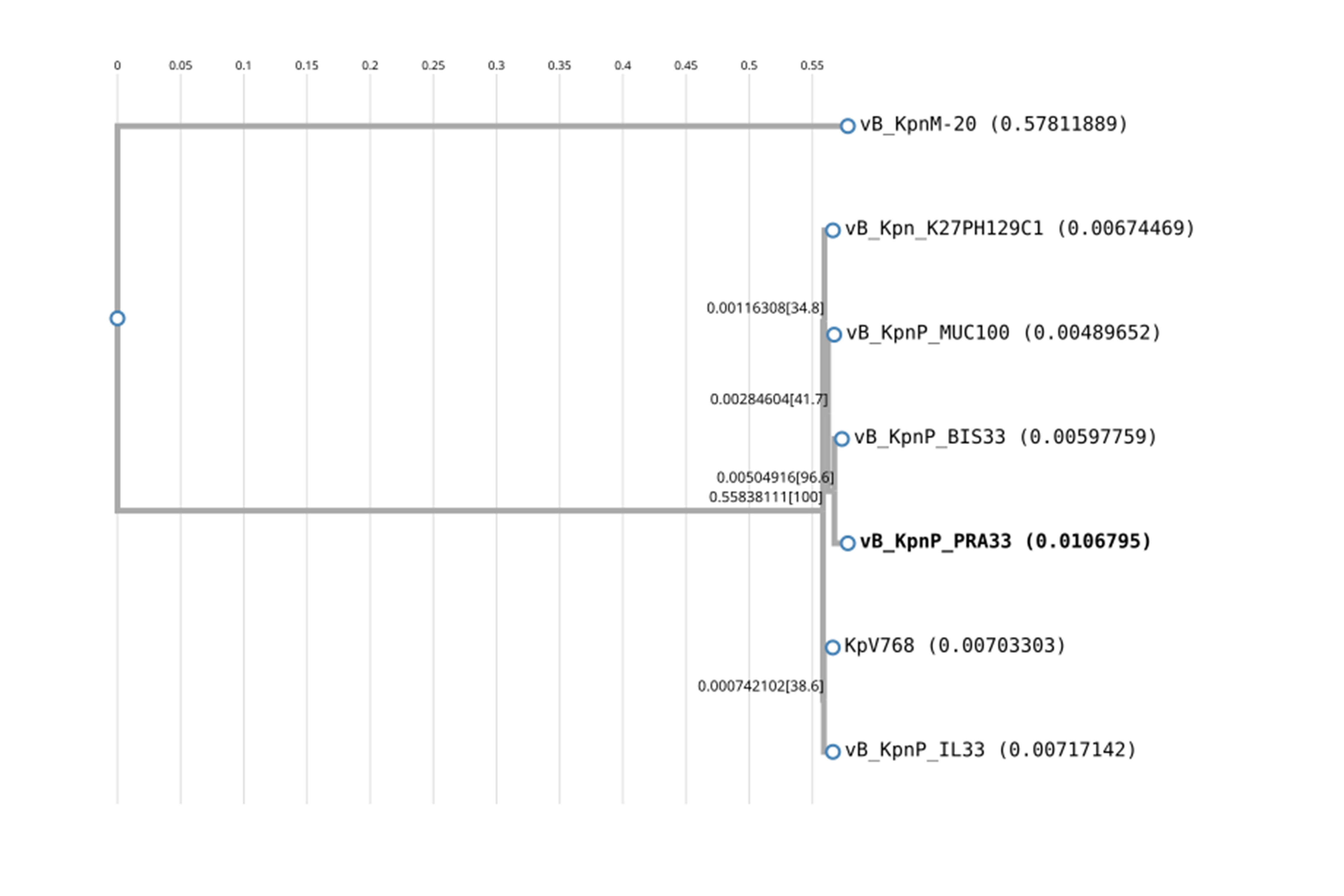

Supplement: Figure S2.TIF [file TEMI_A_2645857_SM5218.tif]

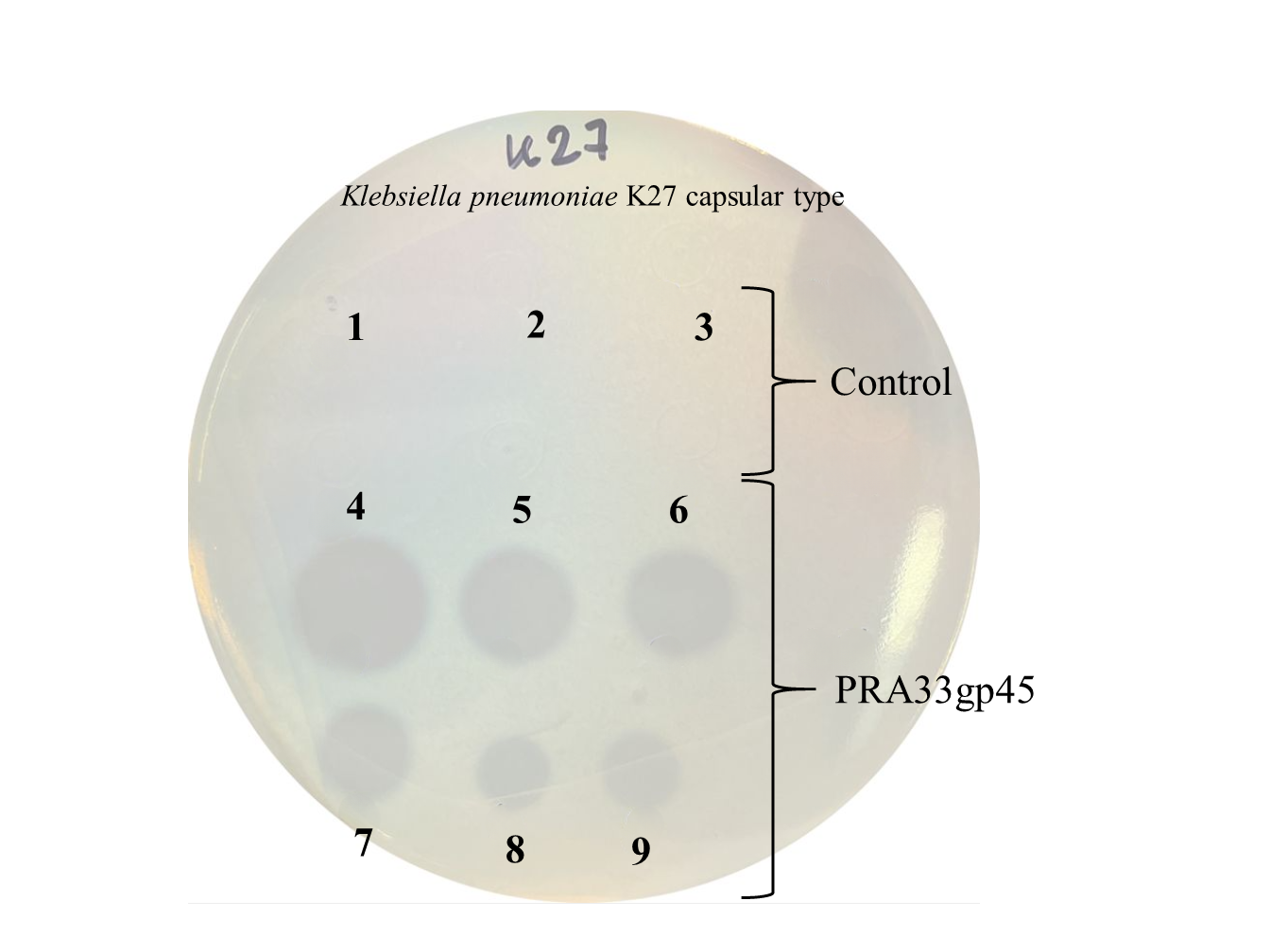

Supplement: Figure S3.TIF [file TEMI_A_2645857_SM5215.tif]
