# Supplementary material for: Phage-derived depolymerase targeting the K27 capsule impairs Klebsiella pneumoniae virulence, biofilm formation, and promotes immune clearance
Source: Emerg Microbes Infect. 2026 Mar 13;15(1):2645857. doi: 10.1080/22221751.2026.2645857 (PMC13063336; doi:10.1080/22221751.2026.2645857)
Supplement: Supplementary Methods.docx [file TEMI_A_2645857_SM5224.docx]

***Molecular Cloning***

*Orf45* (spanning nucleotides 33,404 to 37,132 in the PRA33 phage genome) was amplified by PCR using primers listed in Table S3 (Eurofins genomic, Ebersberg, Germany), purified phage PRA33 genomic DNA as the template, and Phusion polymerase (ThermoFisher Scientific) following the manufacturer’s instructions. The resulting PCR product was purified (Clean-up kit, A&A Biotechnology), digested with appropriate restriction enzymes (FastDigest, ThermoFisher Scientific), and ligated using T4 DNA ligase (ThermoFisher Scientific) into the EcoRI/XhoI sites of the pET28(+) expression vector (Novagen, Merck KGaA, Darmstadt, Germany). Ligation products were transformed into chemically competent *E. coli* Top10F′ cells and plated on LB agar supplemented with kanamycin (50 µg/ml final concentration). Recombinant plasmids were isolated from selected colonies and sequenced (Eurofins genomic) to confirm the in-frame fusion of the expressed protein with the N-terminal His-tag. Similarly, *orf39* (nt 23,053–23,631), *orf40* (nt 23,654–26,029) and *orf44* (nt 29,375–33,340) were cloned into the pET28(+) vector. In all cases, the expressed proteins were fused to an N-terminal His-tag.

***Isolation of capsule polysaccharides from K. pneumoniae A31_1***

Capsule polysaccharides (CPS) were isolated from 5-day old supernatants of stationary-phase K. pneumoniae A31_1 cultures, following the protocol described by Majkowska-Skrobek et al. (2016). The supernatant was incubated with 36.5 % formaldehyde at 25 °C with shaking at 180 rpm for 1 hour. Subsequently, 1 M NaOH was added, and the mixture was incubated for an additional 3 hours at room temperature under continuous agitation. CPS were then collected by centrifugation (15,000 × *g*, 1 hour, 4 °C). After the addition of 20 % (w/v) trichloroacetic acid (TCA), proteins and nucleic acids were removed by centrifugation (16,000 × *g*, 1 hour, 4 °C). The resulting supernatant was precipitated overnight at −20 °C using 1.5 volumes of cold 96 % ethanol. The CPS pellet was recovered by centrifugation (16,000 × *g*, 1 hour, 4 °C), resuspended in Milli-Q water, and dialyzed against water using a 3.5 kDa molecular weight cut-off (MWCO) membrane (SpectraPor, LaboPlus, Warsaw., Poland). Isolated CPS were stored at −20 °C.

***Zymogram assay***

For zymogram assay, purified PRA33gp45 samples (1, 5, and 10 µg) were mixed with non-reducing loading buffer (100 mM Tris-Cl, 20 % (v/v) glycerol, 0.2 % bromophenol blue; pH 8) and incubated on ice for 30 minutes. Samples were then boiled for 5 minutes and loaded onto an 8 % SDS-PAGE gel containing 45 % (v/v) isolated K. pneumoniae A31_1 CPS. After electrophoresis at 4 °C, the gel was washed four times with Milli-Q water at room temperature and incubated in renaturation buffer (20 mM Tris-Cl, 10 mM MgCl₂, 0.1 % (w/v) Triton X-100; pH 7.0) for 72 hours at room temperature with gentle agitation (50 rpm). Following renaturation, the gel was rinsed with Milli-Q water and stained with methylene blue (0.1 % (v/v) in 0.01 % (w/v) KOH) for 1 hour. Destaining with water was continued until clear lytic bands became visible.

***Cytotoxicity assessment of PRA33-derived depolymerase on human lung epithelial cells***

For the cytotoxicity assay, A549 human lung epithelial cells were seeded into two 96-well plates at a density of 0.5 × 10⁴ cells per well in 200 μL of F-12K culture medium. After 48 hours of incubation, the culture medium was replaced with 100 μL of test PRA33gp45 protein solutions (final concentration 500 nM or 250 nM) or a negative control preparation (complete medium supplemented with 5 % FBS and 0.1 % saponin). Plates were then incubated at 37 °C in a humidified atmosphere containing 5 % CO₂ for 24 or 48 hours. Cell viability was assessed using the Cell Proliferation Kit I (MTT, Roche, Sigma-Aldrich) according to the manufacturer’s instructions. Absorbance was measured at 600 nm using a Tecan Sunrise microplate spectrophotometer. Each condition was tested in six repetitions.

***Visualization of biofilms by Confocal microscopy (SCLM)***

*K. pneumoniae* A31_1 (OD_600_ = 0.2) mixed with 70, 140, or 700 nM PRA33gp45 was seeded in glass-bottom plates (Nest, Wuxi, China) and incubated for 20 hours. After washing with 10 mM MgSO_4_, biofilms were stained with 0.001 % acridine orange for 30 minutes, fixed with 2 % formaldehyde, and visualized by confocal microscopy (Laboratory of Confocal Microscopy, University of Warsaw). Numerical analysis was performed using Comstat 2.0 software [Heydorn et al., 2000].

***K. pneumoniae capsule staining***

Cells were grown to OD_600_ = 0.4, centrifuged, and resuspended in F-12K medium to OD_600_ 1.7–1.75. The suspension was treated with 700 nM purified PRA33gp45 (final concentration) and incubated for 30, 60, or 90 minutes at 37 °C. Cells were centrifuged, stained following Maneval’s protocol [Hughes and Smith, 2007], and observed under a 100× oil immersion microscope. Bacterial capsule thickness was measured using ImageJ software [Schneider et al. 2012].

***Visualization of Klebsiella infecting epithelial cells by SCLM***

A549 cells were grown on cover glasses (VWR International, Radnor, PA, USA) placed in 35 mm culture dishes (Nest, Wuxi, China) for 48 hours and then infected with *K. pneumoniae* for 2 h with MOI of ~30. Briefly, after infection, samples were washed with PBS and fixed with 3.7 % formaldehyde for 10 minutes at room temperature. Then after washing, cells were permeabilized with 0.1 % Triton, washed again, and incubated with 2 % BSA for 1 h at room temperature. Then, samples were incubated with Anti-Klebsiella antibodies (PA1-73175 Klebsiella Polyclonal Antibody, Live Technologies, Warsaw, Poland) (diluted 1:25 in 0.1 % BSA) for 18 h, at 4 °C. Next, samples were washed with PBS and incubated with 0.77 µM Phalloidin, Tetramethylrhodamine B isothiocyanate (SIGMA, Merck, Darmstadt, Germany) for 40 minutes at room temperature, followed by washing and DNA staining by 0.5 µg/ml Hoechst 33342 (SIGMA, Merck, Darmstadt, Germany) for 10 minutes at room temperature. Then samples were visualized using a Nikon Eclipse Ti (A1) microscope (Nikon Corporation, Tokyo, Japan) at Laboratory of Electron and Confocal Microscopy, Faculty of Biology, University of Warsaw. Horizontal optical thin sections were collected at 0.21 μm intervals from the outer surface of the biofilm to the bottom of the glass plate. NIS-ELEMENTS interactive software was used for capture and three-dimensional reconstructions of images (Nikon Corporation, Tokyo, Japan). *K. pneumoniae* and host cells were visualized using 3D scanning of infected epithelial cells. Image stacks were acquired at 1 μm Z-axis intervals, enabling visualization of spatial relationships between bacteria, cytoskeletal actin, and nuclei as human cell markers.

1. Majkowska-Skrobek G, et al. Capsule-Targeting Depolymerase, Derived from *Klebsiella* KP36 Phage, as a Tool for the Development of Anti-Virulent Strategy. Viruses. 2016;8(12):324. doi:10.3390/V8120324
2. Heydorn A, et al. Quantification of biofilm structures by the novel computer program COMSTAT. Microbiology (N Y). 2000;146(10):2395–2407. doi:10.1099/00221287-146-10-2395
3. Hughes RB, Smith AC. Capsule Stain Protocols. American Society for Microbiology; 2007. Available from: https://asm.org/ASM/media/Protocol-Images/Capsule-Stain-Protocols.pdf
4. Schneider C, Rasband W, Eliceir, K. NIH Image to ImageJ: 25 years of image analysis. *Nature Methods*. 2012; 9(7), 671–675. doi:10.1038/nmeth.2089
