# Supplementary material for: Phage-derived depolymerase targeting the K27 capsule impairs Klebsiella pneumoniae virulence, biofilm formation, and promotes immune clearance
Source: Emerg Microbes Infect. 2026 Mar 13;15(1):2645857. doi: 10.1080/22221751.2026.2645857 (PMC13063336; doi:10.1080/22221751.2026.2645857)
Supplement: Table S1 S2.docx [file TEMI_A_2645857_SM5222.docx]

Table S1. Bacterial strains used to determine PRA33gp45 specificity

| **Species** | **Strain** | **Plaque with halo** |
| --- | --- | --- |
| *Escherichia coli* | - A4 - A7-3 | - |
| *Staphylococcus aureus* | - A54 - A18_1 - MRSA A106 | - |
| *Pseudomonas aeruginosa* | - A21_2 - A30_2 | - |
| *Klebsiella pneumoniae* | - A31_1 - A38_3 - A99_1 - A101_5 | +  -  -  - |
| *Enterobacter faecalis* | - A66_2 | - |
| *Acinetobacter* | - A86_2 | - |
| *Enterobacter cloace* | - A110 | - |

Table S2. *Klebsiella* spp. used to determine PRA33gp45 specificity

| **K-type** | **Species** | **Strain name** | **Origin** | **Plaque with halo** |
| --- | --- | --- | --- | --- |
| **K1** | *Klebsiella pneumoniae* | **52.144** | Collection de l'Institut Pasteur (CIP) | - |
| **K1** | *Klebsiella pneumoniae* | **NCTC 5054** | National Collection of Type Cultures (NCTC) | - |
| **K2** | *Klebsiella pneumoniae* | **52.145** | Collection de l'Institut Pasteur (CIP) | - |
| **K3** | *Klebsiella pneumoniae* | **271** | Clinical strain (UWr) | - |
| **K3** | *Klebsiella pneumoniae* | **52.146** | Collection de l'Institut Pasteur (CIP) | - |
| **K4** | *Klebsiella ozaenae* | **52.211 T** | Collection de l'Institut Pasteur (CIP) | - |
| **K5** | *Klebsiella ozaenae* | **52.212** | Collection de l'Institut Pasteur (CIP) | - |
| **K6** | *Klebsiella ozaenae* | **52.213** | Collection de l'Institut Pasteur (CIP) | - |
| **K7** | *Klebsiella pneumoniae* | **52.205** | Collection de l'Institut Pasteur (CIP) | - |
| **K8** | *Klebsiella pneumoniae* | **52.206** | Collection de l'Institut Pasteur (CIP) | - |
| **K9** | *Klebsiella pneumoniae* | **52.207.1** | Collection de l'Institut Pasteur (CIP) | - |
|  |  | **52.207.2** | Collection de l'Institut Pasteur (CIP) | - |
| **K10** | *Klebsiella pneumoniae* | **52.214** | Collection de l'Institut Pasteur (CIP) | - |
| **K11** | *Klebsiella pneumoniae* | **52.215** | Collection de l'Institut Pasteur (CIP) | - |
| **K12** | *Klebsiella pneumoniae* | **52.216** | Collection de l'Institut Pasteur (CIP) | - |
| **K13** | *Klebsiella pneumoniae* | **52.217** | Collection de l'Institut Pasteur (CIP) | - |
| **K14** | *Klebsiella pneumoniae* | **52.218** | Collection de l'Institut Pasteur (CIP) | - |
| **K15** | *Klebsiella pneumoniae* | **52.21** | Collection de l'Institut Pasteur (CIP) | - |
| **K16** | *Klebsiella pneumoniae* | **52.220** | Collection de l'Institut Pasteur (CIP) | - |
| **K17** | *Klebsiella pneumoniae* | **52.221** | Collection de l'Institut Pasteur (CIP) | - |
| **K18** | *Klebsiella pneumoniae* | **52.222** | Collection de l'Institut Pasteur (CIP) | - |
| **K19** | *Klebsiella pneumoniae* | **52.223** | Collection de l'Institut Pasteur (CIP) | - |
| **K20** | *Klebsiella pneumoniae* | **52.224** | Collection de l'Institut Pasteur (CIP) | - |
| **K21** | *Klebsiella pneumoniae* | **52.225** | Collection de l'Institut Pasteur (CIP) | - |
|  |  | **52.968** | Collection de l'Institut Pasteur (CIP) | - |
|  |  | **52.358** | Collection de l'Institut Pasteur (CIP) | - |
| **K22** | *Klebsiella pneumoniae* | **52.226** | Collection de l'Institut Pasteur (CIP) | - |
| **K23** | *Klebsiella pneumoniae* | **52.228** | Collection de l'Institut Pasteur (CIP) | - |
| **K24** | *Klebsiella pneumoniae* | **52.229** | Collection de l'Institut Pasteur (CIP) | - |
| **K25** | *Raoultella planticola* | **52.230** | Collection de l'Institut Pasteur (CIP) | - |
| **K26** | *Klebsiella pneumoniae* | **CIP 53.6** | Collection de l'Institut Pasteur (CIP) | - |
| **K27** | *Klebsiella pneumoniae* | **CIP 52.232** | Collection de l'Institut Pasteur (CIP) | + |
| **K28** | *Klebsiella pneumoniae* | **CIP 52.233** | Collection de l'Institut Pasteur (CIP) | - |
| **K29** | *Klebsiella pneumoniae* | **CIP 52.234** | Collection de l'Institut Pasteur (CIP) | - |
| **K30** | *Klebsiella pneumoniae* | **CIP 52.235** | Collection de l'Institut Pasteur (CIP) | - |
| **K31** | *Klebsiella pneumoniae* | **CIP 52.231** | Collection de l'Institut Pasteur (CIP) | - |
| **K32** | *Klebsiella pneumoniae* | **CIP 53.7** | Collection de l'Institut Pasteur (CIP) | - |
| **K33** | *Klebsiella pneumoniae* | **CIP 53.8** | Collection de l'Institut Pasteur (CIP) | - |
| **K34** | *Klebsiella pneumoniae* | **CIP 53.9** | Collection de l'Institut Pasteur (CIP) | - |
| **K35** | *Klebsiella pneumoniae* | **CIP 53.10** | Collection de l'Institut Pasteur (CIP) | - |
| **K36** | *Klebsiella pneumoniae* | **CIP 53.11** | Collection de l'Institut Pasteur (CIP) | - |
| **K37** | *Klebsiella pneumoniae* | **CIP 53.12** | Collection de l'Institut Pasteur (CIP) | - |
| **K38** | *Klebsiella pneumoniae* | **CIP 53.13** | Collection de l'Institut Pasteur (CIP) | - |
| **K39** | *Klebsiella pneumoniae* | **CIP 53.14.1** | Collection de l'Institut Pasteur (CIP) | - |
|  |  | **CIP 53.14.2** | Collection de l'Institut Pasteur (CIP) | - |
|  |  | **CIP 53.14.3** | Collection de l'Institut Pasteur (CIP) | - |
| **K40** | *Klebsiella pneumoniae* | **CIP 53.15** | Collection de l'Institut Pasteur (CIP) | - |
| **K41** | *Klebsiella pneumoniae* | **CIP 53.16** | Collection de l'Institut Pasteur (CIP) | - |
| **K42** | *Klebsiella pneumoniae* | **CIP 53.17** | Collection de l'Institut Pasteur (CIP) | - |
| **K43** | *Klebsiella pneumoniae* | **CIP 53.19** | Collection de l'Institut Pasteur (CIP) | - |
| **K44** | *Klebsiella pneumoniae* | **CIP 53.20** | Collection de l'Institut Pasteur (CIP) | - |
| **K45** | *Klebsiella pneumoniae* | **CIP 53.21** | Collection de l'Institut Pasteur (CIP) | - |
| **K46** | *Klebsiella pneumoniae* | **CIP 53.22** | Collection de l'Institut Pasteur (CIP) | - |
| **K47** | *Klebsiella pneumoniae* | **CIP 53.23** | Collection de l'Institut Pasteur (CIP) | - |
| **K48** | *Klebsiella pneumoniae* | **CIP 53.24** | Collection de l'Institut Pasteur (CIP) | - |
| **K49** | *Klebsiella pneumoniae* | **CIP 52.199** | Collection de l'Institut Pasteur (CIP) | - |
| **K50** | *Klebsiella pneumoniae* | **CIP 52.200** | Collection de l'Institut Pasteur (CIP) | - |
| **K51** | *Klebsiella pneumoniae* | **CIP 52.201** | Collection de l'Institut Pasteur (CIP) | - |
| **K52** | *Klebsiella pneumoniae* | **CIP 53.25** | Collection de l'Institut Pasteur (CIP) | - |
| **K53** | *Klebsiella pneumoniae* | **CIP 53.26** | Collection de l'Institut Pasteur (CIP) | - |
| **K54** | *Klebsiella pneumoniae* | **CIP 53.27** | Collection de l'Institut Pasteur (CIP) | - |
| **K55** | *Klebsiella pneumoniae* | **NCTC 9175** | National Collection of Type Cultures (NCTC) | - |
| **K56** | *Klebsiella pneumoniae* | **NCTC 9176** | National Collection of Type Cultures (NCTC) | - |
| **K57** | *Klebsiella pneumoniae* | **NCTC 9177** | National Collection of Type Cultures (NCTC) | - |
| **K58** | *Klebsiella pneumoniae* | **NCTC 9178.1** | National Collection of Type Cultures (NCTC) | - |
|  |  | **NCTC 9178.2** | National Collection of Type Cultures (NCTC) | - |
| **K59** | *Raoultella planticola* | **NCTC 9179** | National Collection of Type Cultures (NCTC) | - |
| **K60** | *Klebsiella pneumoniae* | **NCTC 9180** | National Collection of Type Cultures (NCTC) | - |
| **K61** | *Klebsiella pneumoniae* | **NCTC 9181.1** | National Collection of Type Cultures (NCTC) | - |
|  |  | **NCTC 9181.2** | National Collection of Type Cultures (NCTC) | - |
| **K61** | *Klebsiella pneumoniae* | **CIP 108293.1** | Collection de l'Institut Pasteur (CIP) | - |
|  |  | **CIP 108283.2** | Collection de l'Institut Pasteur (CIP) | - |
| **K62** | *Klebsiella pneumoniae* | **NCTC 9182** | National Collection of Type Cultures (NCTC) | - |
| **K63** | *Klebsiella pneumoniae* | **NCTC 9183.1** | National Collection of Type Cultures (NCTC) | - |
|  |  | **NCTC 9183.2** | National Collection of Type Cultures (NCTC) | - |
| **K64** | *Klebsiella pneumoniae* | **NCTC 9184** | National Collection of Type Cultures (NCTC) | - |
|  |  | **CIP 80.47** | Collection de l'Institut Pasteur (CIP) | - |
| **K65** | *Raoutella terrigena* | **NCTC 9185** | National Collection of Type Cultures (NCTC) | - |
| **K66** | *Klebisella oxytoca* | **NCTC 9186** | National Collection of Type Cultures (NCTC) | - |
| **K67** | *Klebsiella pneumoniae* | **NCTC 9187** | National Collection of Type Cultures (NCTC) | - |
| **K68** | *Klebsiella pneumoniae* | **NCTC 9188** | National Collection of Type Cultures (NCTC) | - |
| **K69** | *Klebsiella pneumoniae* | **NCTC 9189** | National Collection of Type Cultures (NCTC) | - |
| **K70** | *Klebisella oxytoca* | **NCTC 10261** | National Collection of Type Cultures (NCTC) | - |
| **K71** | *Klebsiella pneumoniae* | **NCTC 10262** | National Collection of Type Cultures (NCTC) | - |
| **K72** | *Raoutella planticola* | **NCTC 10263** | National Collection of Type Cultures (NCTC) | - |
| **K74** | *Klebisella oxytoca* | **NCTC 11355** | National Collection of Type Cultures (NCTC) | - |
| **K79** | *Klebisella oxytoca* | **NCTC 11356** | National Collection of Type Cultures (NCTC) | - |
| **K80** | *Klebsiella pneumoniae* | **NCTC 11357** | National Collection of Type Cultures (NCTC) | - |
| **K81** | *Klebsiella pneumoniae* | **NCTC 11358** | National Collection of Type Cultures (NCTC) | - |
| **K82** | *Klebsiella pneumoniae* | **NCTC 11359** | National Collection of Type Cultures (NCTC) | - |
| **KL101** | *Klebsiella variicola subsp. variicola* | **INF058** | KASPAH collection | - |
| **KL102** | *Klebsiella pneumoniae* | **INF013** | KASPAH collection | - |
| **KL103** | *Klebsiella variicola subsp. variicola* | **INF152** | KASPAH collection | - |
| **KL105** | *Klebsiella variicola subsp. variicola* | **INF081** | KASPAH collection | - |
| **KL107** | *Klebsiella pneumoniae* | **INF007** | KASPAH collection | - |
| **KL108** | *Klebsiella pneumoniae* | **INF063** | KASPAH collection | - |
| **KL109** | *Klebsiella pneumoniae* | **INF016** | KASPAH collection | - |
| **KL111** | *Klebsiella pneumoniae* | **INF215** | KASPAH collection | - |
| **KL112** | *Klebsiella pneumoniae* | **INF202** | KASPAH collection | - |
| **KL113** | *Klebsiella pneumoniae* | **INF088** | KASPAH collection | - |
| **KL114** | *Klebsiella pneumoniae* | **INF053** | KASPAH collection | - |
| **KL116** | *Klebsiella pneumoniae* | **INF002** | KASPAH collection |  |
| **KL118** | *Klebsiella pneumoniae* | **INF344** | KASPAH collection | - |
| **KL119** | *Klebsiella pneumoniae* | **INF226** | KASPAH collection | - |
| **KL120** | *Klebsiella variicola subsp. variicola* | **INF047** | KASPAH collection | - |
| **KL122** | *Klebsiella pneumoniae* | **INF032** | KASPAH collection | - |
| **KL123** | *Klebsiella quasipneumoniae subsp. similipneumoniae* | **INF195** | KASPAH collection | - |
| **KL124** | *Klebsiella pneumoniae* | **INF184** | KASPAH collection | - |
| **KL125** | *Klebsiella pneumoniae* | **INF065** | KASPAH collection | - |
| **KL127** | *Klebsiella pneumoniae* | **INF097** | KASPAH collection | - |
| **KL130** | *Klebsiella pneumoniae* | **INF090** | KASPAH collection | - |
| **KL131** | *Klebsiella pneumoniae* | **INF092** | KASPAH collection | - |
| **KL132** | *Klebsiella pneumoniae* | **INF118** | KASPAH collection | - |
| **KL133** | *Klebsiella pneumoniae* | **INF132** | KASPAH collection | - |
| **KL134** | *Klebsiella pneumoniae* | **INF149** | KASPAH collection | - |
| **KL137** | *Klebsiella variicola subsp. variicola* | **INF112** | KASPAH collection | - |
| **KL139** | *Klebsiella pneumoniae* | **INF054** | KASPAH collection | - |
| **KL140** | *Klebsiella pneumoniae* | **INF156** | KASPAH collection | - |
| **KL141** | *Klebsiella pneumoniae* | **INF188** | KASPAH collection | - |
| **KL142** | *Klebsiella pneumoniae* | **INF057** | KASPAH collection | - |
| **KL143** | *Klebsiella pneumoniae* | **INF200** | KASPAH collection | - |
| **KL144** | *Klebsiella quasipneumoniae subsp. similipneumoniae* | **INF207** | KASPAH collection | - |
| **KL146** | *Klebsiella pneumoniae* | **INF117** | KASPAH collection | - |
| **KL153** | *Klebsiella pneumoniae* | **INF321** | KASPAH collection | - |
| **KL154** | *Mixed* | **INF323** | KASPAH collection | - |
| **KL158** | *Klebsiella pneumoniae* | **INF023** | KASPAH collection | - |
| **KL163** | *Klebsiella pneumoniae* | **0967/02/08** | Clinical strain (UWr) | - |
| **KL166** | *Klebsiella pneumoniae* | **INF066** | KASPAH collection | - |
| **KL167** | *Klebsiella pneumoniae* | **INF062** | KASPAH collection | - |
| **KL168** | *Klebsiella pneumoniae* | **INF137** | KASPAH collection | - |
| **KL169** | *Klebsiella pneumoniae* | **INF128** | KASPAH collection | - |
| **KL170** | *Klebsiella quasipneumoniae subsp. similipneumoniae* | **INF309** | KASPAH collection | - |
