# Supplementary material for: Phage-derived depolymerase targeting the K27 capsule impairs Klebsiella pneumoniae virulence, biofilm formation, and promotes immune clearance
Source: Emerg Microbes Infect. 2026 Mar 13;15(1):2645857. doi: 10.1080/22221751.2026.2645857 (PMC13063336; doi:10.1080/22221751.2026.2645857)
Supplement: Supplementary Results.docx [file TEMI_A_2645857_SM5221.docx]

**PRA33gp45 exhibits no cytotoxicity toward human lung epithelial cells**

Cytotoxicity of PRA33gp45 against human A549 cells under the conditions previously employed to study *K. pneumoniae* interactions with these cells was evaluated.. Dilution of culture medium slightly decrease cell viability as compared to untreated human cells. Neither 700 nM nor 350 nM concentrations of PRA33gp45 had any negative influence on cell viability after 24 or 48 hours exposition (Figure S4). In contrast, the negative control (0.1% saponin) demonstrated marked cytotoxicity at both time points, confirming the validity of the assay.
