# Supplementary material for: Phage-derived depolymerase targeting the K27 capsule impairs Klebsiella pneumoniae virulence, biofilm formation, and promotes immune clearance
Source: Emerg Microbes Infect. 2026 Mar 13;15(1):2645857. doi: 10.1080/22221751.2026.2645857 (PMC13063336; doi:10.1080/22221751.2026.2645857)
Supplement: Table S4 S5.docx [file TEMI_A_2645857_SM5217.docx]

Table S4. Product sizes of four serotyping PCR reactions

| primer pair | primers | product size |
| --- | --- | --- |
| 1 | KP-wza-CF1 + KP-wzc-CR1 | 2.7 kb |
| 2 | KP-wza-CF2 + KP-wzc-CR1 | 3.4 kb |
| 3 | KP-wza-CF1 + KP-wzc-CR2 | 2.4 kb |
| 4 | KP-wza-CF2 + KP-wzc-CR2 | 3.1 kb |

Table S5. Analysis of PCR products by BLASTn

| PCR product | reference region coverage [%] | coverage identity to reference region [%] |
| --- | --- | --- |
| 1 | 73 | 97.96 |
| 2 | 85 | 94.43 |
| 3 | 80 | 98.53 |
| 4 | 86 | 98.31 |
