# Supplementary material for: Phage-derived depolymerase targeting the K27 capsule impairs Klebsiella pneumoniae virulence, biofilm formation, and promotes immune clearance
Source: Emerg Microbes Infect. 2026 Mar 13;15(1):2645857. doi: 10.1080/22221751.2026.2645857 (PMC13063336; doi:10.1080/22221751.2026.2645857)
Supplement: Table S3.docx [file TEMI_A_2645857_SM5216.docx]

Table S3. PCR primers used in this study

| Primer name | sequence | Cloning site | Target | PCR product size bp |
| --- | --- | --- | --- | --- |
| PRA33_45F | GCGAATTCGCGGACCAAGACATTAAAACAGTCATTCAGTA | EcoRI | *orf*45 | 3745 |
| PRA33_45R | GGCTCGAGCTATTTGTTTAGCAGAAGCTCCTCAAG | XhoI |  |  |
| PRA33_44F | GCAAGCTTTGAGCTACGACAAGTCCAAACCTAGTGACTAC | EcoRI | *orf*44 | 3966 |
| PRA33_44R | GGCTCGAGTTAGTTGGGTTTAGGCTGCTGTTTGATGGTTAC | XhoI |  |  |
| PRA33_40F | GCGAATTCGCTCTCGTATCACAGTCAATCAAAAATCTTAAG | EcoRI | *orf*40 | 2389 |
| PRA33_40R | GGCTCGAGTTAAATACCGTTAGCGCGTCTCATATAG | XhoI |  |  |
| PRA33_39F | GCAAGCTTTGAACATGCAAGATGCTTACTTTGGGTCTGCCGCTGAG | EcoRI | *orf*39 | 603 |
| PRA33_39R | GGCTCGAGTTAACGACCGATGAGACCCTGCACGTATGCGTCAC | XhoI |  |  |
| KP-wza-CF1 | TGAAAGTGTTTGTCATGGG | none | *wza* |  |
| KP-wza-CF2 | GGGTTTTTATCGGGTTGTAC |  |  |  |
| KP-wzc-CR1 | TTCAGCTGGATTTGGTGG |  | *wzc* |  |
| KP-wzc-CR2 | GCTTCCATCATTGCAAAATG |  |  |  |
